# Completion of the Transfer of the Online Journal of Public Health Informatics (OJPHI) to JMIR Publications

**DOI:** 10.2196/50243

**Published:** 2023-07-18

**Authors:** Edward Mensah

**Affiliations:** 1 School of Public Health University of Illinois Chicago Chicago, IL United States

**Keywords:** public health informatics, data science, precision public health, artificial intelligence, health promotion, disease prevention

## Abstract

Founded in 2009, the *Online Journal of Public Health Informatics* (OJPHI) strives to provide an unparalleled experience as the platform of choice to advance public and population health informatics. As a premier peer-reviewed journal in this field, OJPHI’s mission is to serve as an advocate for the discipline through the dissemination of public health informatics research results and best practices among practitioners, researchers, policymakers, and educators. However, in the current environment, running an independent open access journal has not been without challenges. Judging from the low geographic spread of our current stakeholders, the overreliance on a small volunteer management staff, the limited scope of topics published by the journal, and the long article turnaround time, it is obvious that OJPHI requires a change in direction in order to fully achieve its mission. Fortunately, our new publisher JMIR Publications is the leading brand in this field, with a portfolio of top peer-reviewed journals covering innovation, technology, digital medicine and health services research in the internet age. Under the leadership of JMIR Publications, OJPHI plans to expand its scope to include new topics such as precision public health informatics, the use of artificial intelligence and machine learning in public health research and practice, and infodemiology in public health informatics.

## Introduction

The *Online Journal of Public Health Informatics* (OJPHI) has been delivering the latest developments in the emerging field of public health informatics since 2009. The journal was originally created to fill a gap in the public health informatics publishing and training landscape. I started to recognize the need for such a journal as a result of my own involvement in advancing training and education in public health informatics. In 2002, I had the privilege to cofound and serve as director of the graduate program in Public Health Informatics at the University of Illinois Chicago (UIC) School of Public Health, the very first program of its kind in the United States.

The immeasurable value of transforming raw data into information and knowledge for effective and efficient decision-making by using information and communication technologies (ICTs) was evident during the COVID-19 pandemic. Although public health is an information-intensive field, it lags behind other health-related fields in the utilization of ICTs for the delivery of services and resource management. The emergence of public health informatics as a professional specialty is part of a larger development of informatics in health-related fields, including medicine, nursing, pharmacy, and dentistry. The interest in informatics as a specialty within these fields reflects the significance of data collection, analysis, and transformation into information and knowledge in the health care sector. Several journals have been launched in response to the growing need for informaticians in these health care disciplines, and many of the JMIR Publications journals, not least one of their flagship journals *JMIR Public Health and Surveillance*, also publish research at the intersection of public health and technology.

Unfortunately, the COVID-19 pandemic and the subsequent increased interest and research output in our discipline also demonstrated the limitations of a self-published, open access journal relying largely on volunteer efforts. Not only is it becoming increasingly challenging to find peer reviewers, but it is also increasingly difficult to operate in a rapidly changing and complex scholarly publications landscape while running on a shoestring budget. With a broader shift toward open access, libraries now often direct their publications budget toward transformative (hopefully transitional!) agreements that support costs to publish in former subscription journals of large publishing corporations and phasing out institutional open access funds, leaving little or no support for independent journals.

Considering all these factors, it is now time to put the future of OJPHI into professional hands, by choosing a mission-driven publisher that has its roots in academia. JMIR Publications, a medium-sized but rapidly growing publisher with its mission-driven academic leadership and focus on innovation in health and medicine, is the ideal fit as a new home and publisher of OJPHI. With access to the experienced professional staff of JMIR Publications, OJPHI will attract stakeholders from a wide variety of disciplines and geographic areas, achieve a shorter turnaround time, and upgrade the quality of the journal. The new publisher also has the capacity to make deals with institutional partners to put the journal on a more financially stable basis.

Like other journals in the JMIR Publications portfolio, OJPHI seeks to promote interdisciplinary collaboration and welcomes contributions by researchers and practitioners from a wide range of fields, including public health, computer science, data science, health informatics, and related disciplines. As such, it is complementary to the over 34 other titles in the JMIR Publications portfolio, and authors will have the opportunity to transfer their submissions between journals without the need for a new peer review (portable peer reviews).

OJPHI invites submissions of original research articles, reviews, and perspectives or viewpoints that cover a broad range of topics related to public health informatics, including the following:

- Use of health ICTs and data science to improve public health
- Development and implementation of electronic health records and other health information systems by public health agencies
- Framework, evaluation, and use of health information exchange technologies among public health agencies, hospitals, laboratories, and clinics
- Use of data analytics (including artificial intelligence [AI], machine learning, geographic information system, visualization, and data mining technologies) in public health research and practice
- Use of social media in public and population health informatics applications for health promotion and disease prevention
- Evaluation of mobile health technology and digital platforms in public health practice
- Integration of social determinants of health within public health practice using ICTs
- Ethical, legal, and social implications of public health informatics
- Precision public health informatics
- Analysis and utilization of big data for health promotion and health equity
- Development and evaluation of contact tracing technologies for public health practice and policy
- Infodemiology in public health informatics (complementing similar sections in the *Journal of Medical Internet Research*, *JMIR Public Health and Surveillance*, and the recently launched journal *JMIR Infodemiology*)

The manuscript management system and journal homepage has been migrated to the JMIR Publications platform (under its new URL ojphi.jmir.org) and submissions are now open as of July 2023. Articles under consideration on the current platform are in the process of being migrated, and the review process for the articles in the pipeline will commence thereafter. For new articles, authors are encouraged to indicate their preferences by selecting from the list the topics under which they want their articles to be reviewed. This will aid search engine optimization and make it easier to automate the process of allocating the articles to specific reviewers. Authors of manuscripts submitted to other JMIR Publications journals are also encouraged to consider OJPHI as a destination for manuscript transfer.

In addition, we are refreshing our editorial board, and I invite interested researchers who are passionate about advancing the field of public health informatics to contact us. I am personally excited about the future of the field and that of the journal, which I consider in excellent hands with JMIR Publications. I thank our stakeholders, including authors and peer reviewers, for their continued patience and support as we are making this transition.

Joining JMIR Publications, an established and top-notch publisher, positions OJPHI within a wider ecosystem of open access biomedical and health informatics journals. We now have the facilities needed to reach out to a diverse group of authors and readers from universities, public health agencies, and policy makers in developed countries and the Global South. JMIR Publications provides us with the platform to improve the quality of the journal, article turnaround time, and become the public and population health informatics journal of choice among our stakeholders.

## Abbreviations

AI: artificial intelligence
ICT: information and communication technology
OJPHI: Online Journal of Public Health Informatics
UIC: University of Illinois Chicago

